# HEALTHY AGING IN PLACE REQUIRES A HEALTHY HOME CARE WORKFORCE: THE SAFETY AND HEALTH OF PAID CAREGIVERS

**DOI:** 10.1093/geroni/igac059.628

**Published:** 2022-12-20

**Authors:** Margaret Quinn, Pia Markkanen, Susan Sama, Catherine Galligan, John Lindberg, Rebecca Gore

**Affiliations:** University of Massachusetts Lowell/College of Health Sciences, Lowell, Massachusetts, United States; University of Massachusetts Lowell, Lowell, Massachusetts, United States; University of Massachusetts Lowell, Lowell, Massachusetts, United States; University of Massachusetts Lowell, Lowell, Massachusetts, United States; University of Massachusetts Lowell, Lowell, Massachusetts, United States; University of Massachusetts Lowell, Lowell, Massachusetts, United States

## Abstract

Home care (HC) aides provide health and personal care services that enable older adults to live at home as they age. Aides are increasingly in demand as our population ages rapidly. Yet there is an aide shortage due in part to instabilities in HC work organization and to occupational safety and health (OSH) hazards, resulting in aides losing work time or leaving their jobs. Aide injuries and illness interrupt the continuity of care delivery and HC hazards can put clients and family caregivers at risk. This research synthesizes recent findings of aide OSH studies in order to identify risks and preventive interventions to improve both HC aide and client safety. Mixed methods were used to characterize aide OSH hazards including needlesticks, musculoskeletal strain, experiences of violence, COVID-19 and other infections and harmful disinfection practices. Improving aide OSH can contribute to improved care services enabling older adults to live at home.

